# Acoustic Noise Induces Attention Shifts and Reduces Foraging Performance in Three-Spined Sticklebacks (*Gasterosteus aculeatus*)

**DOI:** 10.1371/journal.pone.0017478

**Published:** 2011-02-28

**Authors:** Julia Purser, Andrew N. Radford

**Affiliations:** School of Biological Sciences, University of Bristol, Bristol, United Kingdom; Institute of Marine Research, Norway

## Abstract

Acoustic noise is known to have a variety of detrimental effects on many animals, including humans, but surprisingly little is known about its impacts on foraging behaviour, despite the obvious potential consequences for survival and reproductive success. We therefore exposed captive three-spined sticklebacks (*Gasterosteus aculeatus*) to brief and prolonged noise to investigate how foraging performance is affected by the addition of acoustic noise to an otherwise quiet environment. The addition of noise induced only mild fear-related behaviours - there was an increase in startle responses, but no change in the time spent freezing or hiding compared to a silent control - and thus had no significant impact on the total amount of food eaten. However, there was strong evidence that the addition of noise increased food-handling errors and reduced discrimination between food and non-food items, results that are consistent with a shift in attention. Consequently, noise resulted in decreased foraging efficiency, with more attacks needed to consume the same number of prey items. Our results suggest that acoustic noise has the potential to influence a whole host of everyday activities through effects on attention, and that even very brief noise exposure can cause functionally significant impacts, emphasising the threat posed by ever-increasing levels of anthropogenic noise in the environment.

## Introduction

The addition of acoustic noise to the environment can significantly affect the well-being of humans and many other animals. While we might expect to see avoidance behaviour in response to deleterious noise, the extent to which an animal can move away from the source will often be limited [1], [2], and it is therefore also crucial to examine behaviour of animals when additional noise is unavoidable [3]. Some of the most obvious effects of additional noise (auditory damage [4]–[8], stress responses [9]–[12], altered signal detection and communication [1], [2]) have received a fair degree of attention in some taxa. However, the possible impact of noise on a whole range of functionally important behaviour is largely unexplored. Foraging activity is a clear example of such behaviour: it is important to all animals, with any disruption likely to have consequences for survival and reproduction, and yet the ways in which it is potentially impacted by noise have seldom been investigated in any taxa [13].

Noise could influence foraging performance by masking critical acoustic information (such as the sounds made by prey [14]), a classic auditory effect of noise exposure. Existing examples of auditory masking include traffic noise interfering with the perception of mating signals in the grey treefrog, *Hyla chrysoscelis* [15], and in a number of urban bird species [16]. However, noise may also affect primarily visual foraging behaviour through cross-modal effects of two kinds. First, noise might act as a stressor with suites of behaviour changing as part of an allostatic response to an environmental event [10], [17]–[19], affecting the intensity, duration or frequency of certain activities. This might include: a reduction or cessation of normal locomotor activity (as seen with predation risk [20]), which would decrease the time spent foraging and could result in a lowered overall food intake; an overall reduction in appetite, mediated by peptides associated with the corticotropin-releasing factor system [21]; or an increase in appetite and food intake, due to moderate increases in the level of cortisol arising from activation of the hypothalamic-pituitary-interrenal axis [22]. Second, because foraging involves various cognitive processes, including detection and classification [23], noise might influence foraging performance in more subtle ways through effects on attention. These effects on attention could occur as part of an allostatic response to a noise stressor, albeit involving alterations to behaviour that are more specific than the general effects on activity levels that we typically expect; alternatively, attention may be affected by noise in the absence of any allostatic stress response, for example, if noise distracts an animal [24] without inducing a stress response. Although the detrimental effects of noise on attention have long been established in humans (reviewed by [25]), this link has received far less consideration in non-human animals (see [26], [27]).

Behaviourally, the addition of noise to an animal's environment might affect attention to the detriment of foraging performance in two main ways. It could cause a narrowing in attention, whereby animals ignore certain secondary stimuli or focus attention over a smaller spatial area (stimulus-selective attention or spatially-selective attention; [28]). This could reduce the foraging search rate, since a given area takes longer to search with a narrow focus [29], or mean that spatially peripheral stimuli will be detected less well [30]. Noise could also induce attention shifts away from the primary foraging task (just as *Spodoptera littoralis* moths ignore olfactory cues when simultaneously exposed to certain acoustic cues [31]). Individuals might focus their attention on searching for the sound source or might be more easily distracted by non-food items. These attention-mediated effects of noise are driven by a limited capacity to attend simultaneously to multiple stimuli [26], and thus might occur when all sorts of sound stimuli are added to an animal's environment, while the more obvious noise effects (auditory damage, masking and allostatic stress-related responses) might only occur in response to noise and sounds of a specific nature.

We investigated the potential impact of acoustic noise on foraging behaviour using the three-spined stickleback (*Gasterosteus aculeatus*), a model fish species that acclimates well to laboratory conditions [32]. While the potential impacts of acoustic noise on fish hearing [7], [8], signal detection and communication [2] and stress responses [11], [12] have received a fair degree of attention, there is a general paucity of research examining how noise affects everyday behaviour of fish, and foraging in particular is largely unexplored in this context [2], [33]. In both captive and wild conditions, sticklebacks are likely to encounter various sources of noise which may potentially disrupt their primarily visual foraging behaviour [34]. By exposing sticklebacks foraging on live prey to both a ‘silent’ control (representing baseline foraging conditions) and to playback of bandwidth-limited white noise, we answer two main questions. First, how is foraging performance impacted by brief and prolonged exposure to noise? Second, to what extent is the observed reduction in foraging performance a result of general allostatic responses or more specific changes in attention?

## Methods

### (a) Ethics statement

Fish showed no signs of adverse reactions to the test set-up. Measures of stress-related behaviour in the study confirm that the fish were not unduly disturbed by the test procedures during control conditions, and showed only mild startle/sensitisation effects during the noise playbacks. Fish all appeared to return to normal pre-trial behaviour when inspected and fed at the end of each test day.

This research adhered to the Association for the Study of Animal Behaviour/Animal Behavior Society Guidelines for the Use of Animals in Research, the legal requirements of the country (UK) in which the work was carried out and all institutional guidelines. The University of Bristol Animal Services Ethical Committee approved the procedures under UIN: UB/09/010.

### (b) Study species and housing

**Figure 1:**
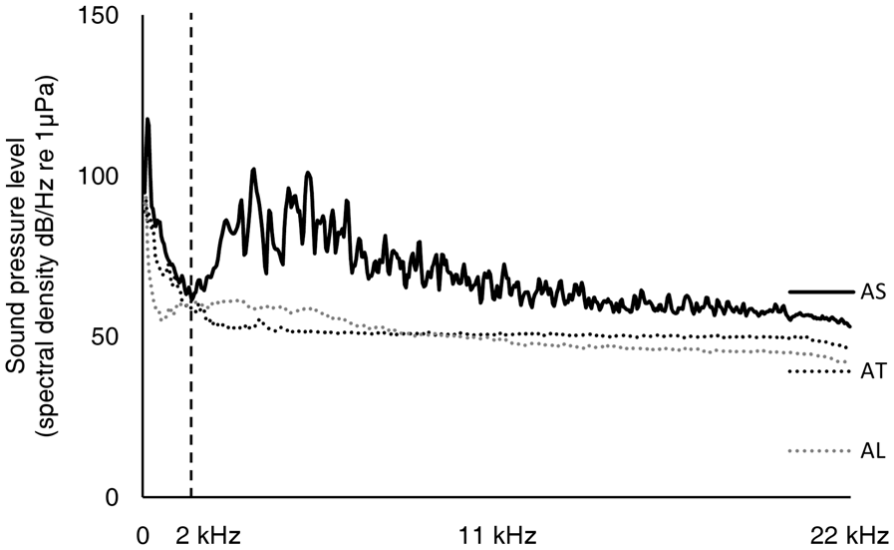
Ambient noise in standard pre-trial housing tanks. Ambient sound pressure levels (spectral density, dB/Hz re 1 µPa) from averaged power spectra (FFT analysis: spectral level units, Hann evaluation window, 50% overlap, FFT size 1024) recorded in standard stock tanks used for housing stickleback prior to studies (AS), ambient conditions in still freshwater test tanks (AT), and ambient conditions in an example freshwater lake in the UK (AL).

Twenty-four adult three-spined sticklebacks were used as subjects, with an additional twelve individuals (familiar to the subjects) used as companion fish; presence of a ‘companion’ fish in proximity to the focal fish aids normal behaviour of the focal fish during testing (stickleback are housed in stable social groups). Fish were wild-caught in a UK river by a reputable biological supplier. Prior to experimental testing, fish were acclimated to the captive environment in the indoor fish facilities at the University of Bristol: groups of up to 20 sticklebacks were housed in 100 l stock tanks with an air sponge and external power filtration, at 10°C on a 12:12 light dark cycle (keeping the fish in non-breeding winter condition), and fish were fed three times per week on frozen bloodworm (*Chironomid* larvae). Care was taken to minimise the intrusion of artificial noise into the stock tanks, particularly with regard to frequencies below 2 kHz, which are in the possible hearing range of fish, such as the three-spined stickleback (see [35] for an audiogram of another Gasterosteidae, the nine-spined stickleback *Pungitius pungitius*), that have a swim bladder but no known hearing “specializations” [36]. Fish were housed in tanks placed on thick polystyrene boards in thermally insulated rooms, within a building separate from the associated University building (minimising the transmission of low-frequency external building noises); external power filters were placed on a separate base (minimising transmission of low-frequency filter noise); power filter outflows were piped underneath the water surface (minimising noisy disruption of the water surface); and sponge filter air flows were at low pressure (minimising low-frequency noise from filter vibration and high-frequency noise from air bubbles). The resulting ambient sound levels, at frequencies below 2 kHz, were slightly higher in standard pre-trial housing tanks than the ambient acoustic conditions in still freshwater test tanks (with no filtration or air flow; median difference of sound pressure level (SPL) in 43 Hz steps from 43–1938 Hz (spectral density, dB/Hz): 7 dB re 1 µPa; Figure 1) and an example freshwater lake habitat (median difference: 9 dB re 1 µPa; Figure 1), but comparable to freshwater streams and rivers reported in [37] that are typical of the habitat where the study species lives naturally.

### (c) Noise treatments

**Figure 2:**
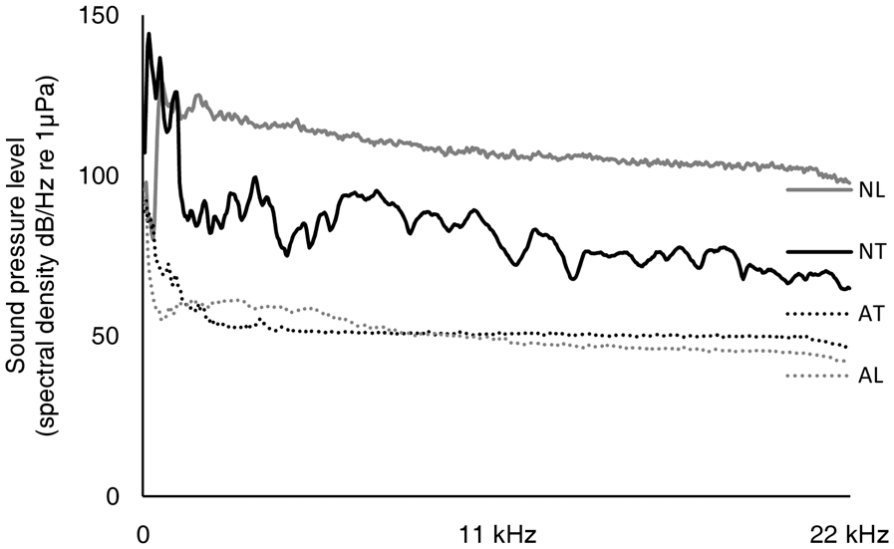
Noise in the feeding areas of the experimental tank during playback and silent control conditions. Sound pressure levels (spectral density, dB/Hz re 1 µPa) from averaged power spectra (FFT analysis: spectral level units, Hann evaluation window, 50% overlap, FFT size 1024) recorded during playback of white noise in experimental test tank (NT) and during ambient silent control conditions (AT). Sound pressure levels from recordings in an example freshwater lake in the UK during recreational speedboat activity (NL) and during ambient conditions (AL).

Each subject experienced three trials - prolonged noise, brief noise, silent control - in a repeated-measures design (treatment order counterbalanced across subjects), with 72 hours between trials to each individual (all trials to the same subject conducted at the same time of day). The silent control gave a baseline measure of foraging performance under ambient conditions (as in, for example, [13] [24]). Noise treatments used white noise, bandwidth limited between 100 and 1000 Hz, presenting frequencies that fall within the likely hearing range of the stickleback. Ecologically speaking, the SPL of the playback noise is comparable to the peak SPLs (between 100 and 1000 Hz) recorded at the shoreline of lakes where recreational speedboats are active (Figure 2 and [38]), well above the ambient noise levels found in a range of freshwater habitats [37], and thus valid for investigating the addition of noise to an otherwise quiet environment. Noise treatments should thus be detectable by the fish, but without reaching SPLs that might cause auditory damage; sensitivity to temporary auditory damage appears to be reduced for fish with no specialist hearing adaptations compared to ‘specialist’ species [8], [39], and the durations of noise exposure in this study were relatively brief.

Sound files were created in Avisoft SASLab Pro v4.52 (Avisoft Bioacoustics), and were played back as WAV files using: wav/mp3 player (TrekStor™ GmbH & Co.; frequency range: 20–20,000 Hz); amplifier (Kemo Electronic GmbH; 18 W; frequency response range: ∼40–20,000 Hz); potentiometer (set to minimum resistance; Omeg Ltd; 10k logarithmic); and Aqua30 underwater speaker (DNH; effective frequency range 80–20,000 Hz; full specification data available at http://www.dnh.no/uploads/filer/365-AQUA30.pdf).

Noise in the feeding areas of the experimental tank during playback and silent control conditions were measured using a hydrophone with preamplifier (High Tech Inc. HTI 96-MIN; manufacturer-calibrated sensitivity −164.3 dB re 1v/µPa; frequency range 2–30,000 Hz) and solid-state recorder (Edirol R09HR, Roland Corporation; 44.1 kHz sample rate; calibrated against reference tone of known amplitude). Averaged power spectra of the tank recordings were calculated in Avisoft using a fast Fourier transform (FFT) analysis (spectrum level units normalized to 1 Hz bandwidth, Hann evaluation window, 50% overlap, FFT size 1024; averaged from 5 s of recording), and are displayed in Figure 2 alongside recordings of an example freshwater lake in the UK during recreational speedboat activity (recorded near the shoreline, approximately 50 cm below water surface, boat passing within 10 m of the hydrophone; power spectrum averaged from 1 s recording when boat nearest, i.e. at peak SPL) and during ambient conditions (power spectrum averaged from 5 s of recording). Due to the acoustic properties of small tanks, the white noise playback featured distinct peaks of sound pressure rather than the original uniform SPL between 100 and 1000 Hz (Figure 2).

### (d) Experimental protocol

**Figure 3:**
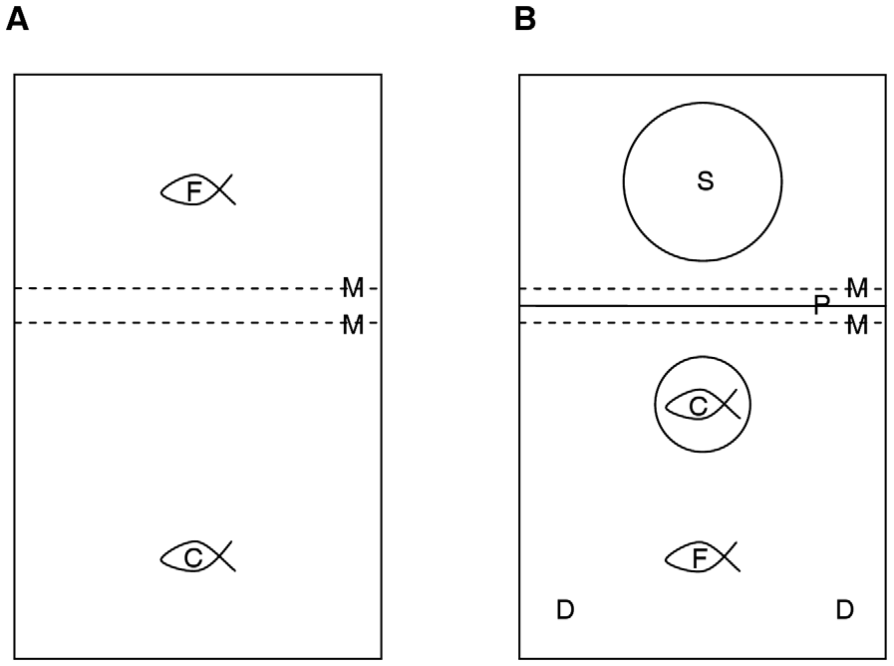
Experimental test tank schematic: arrangement of fish and apparatus during and between trials. Plan view of test tank before and between trials (A) and during trials (B): focal fish (F), companion fish (C) contained in transparent plastic cylinder during trials, two areas where live *Daphnia* sp. were delivered during trials (D), speaker (S) behind opaque partition (P) during trials, mesh partitions (M) separated fish between trials. Fish in separate sections of the tank between trials with mesh partitions allowing visual, acoustic and olfactory contact.

At the start of the study, fish were allowed to acclimate to their test tank: 20 h before the first trial began; fish were transferred in focal-fish/companion-fish pairs from their stock tank to separate sections of a 10 l test tank, with a mesh divider to allow social contact (Figure 3a). For trials, the focal fish and companion fish were both in the same section of the tank (Figure 3b), with the companion fish contained within a transparent plastic cylinder (diameter: 7 cm): the companion fish was first confined within the transparent cylinder and then the focal fish was transferred to the adjacent open space. An opaque partition was added between the two tank sections, to allow subsequent addition of the speaker to the tank out of sight of the fish. Trials were conducted in 12 parallel tanks with 12 sequential trials (one trial per fish) per session; a single speaker was used for all trials with the speaker being moved between the12 tanks used in each testing session. Fish were left to acclimate for 1 h before the testing session began. At the beginning of each trial the speaker was added to the tank, behind the opaque partition, and the fish allowed to settle; they resumed normal swimming behaviour within 2–3 min after the introduction of the speaker. This movement of the focal fish between sections of the tank allowed the most efficient transfer from pre-trial to trial conditions, with focal fish and companion fish separated and identifiable, and thus the least disturbance to the fish. Between trials, fish were returned to the pre-trial conditions (Figure 3a): the speaker was removed, focal fish transferred back to the second tank section, companion fish released from the transparent compartment, and the opaque partition between tank sections removed.

**Figure 4:**
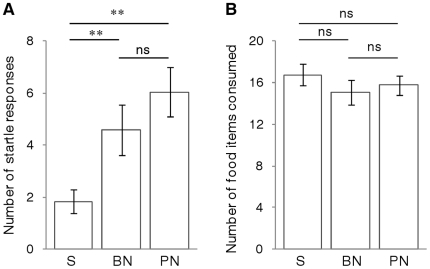
Acoustic noise increases startle responses but does not affect total food consumption. Response of foraging sticklebacks to playbacks of silence (S), brief (10 s) white noise (BN) and prolonged (300 s) white noise (PN). Bars show mean±1s.e.m. response for 24 fish during each playback of a repeated-measures experiment, with significant (** p≤0.01) and non-significant (ns p≥0.05) posthoc differences indicated (paired t-tests with Bonferroni correction). Brief noise and prolonged noise both significantly affected (A) the number of startle responses, but had no significant effect on (B) the total number of food items consumed.

During trials, live *Daphnia* sp. were delivered by hand to each side of the tank to provide a distributed food source. *Daphnia* delivery was conducted in a standard manner in all trials, using Pasteur pipettes pre-filled with a suspension of numerous live *Daphnia*. Pipettes were moved towards the tank in a smooth manner until the tip was approximately 15 mm above the water surface in one of two corners of the tank nearest the experimenter. Pipettes were then squeezed until one *Daphnia* (or two at the start of trials) dropped gently into the water. Pipettes were then moved away from the tank until they were below the line of sight of the tank (and swapped for full pipettes as necessary; kept within reach so that no experimenter body movement was required). Since fish are normally fed with researchers in full view and observed closely for husbandry purposes, and are thus well-acclimated to human proximity, *Daphnia* delivery during these trials was conducted with the experimenter in full sight of the fish. Fish showed no signs of adverse reaction to this *Daphnia* delivery method during pre-trial tests, and startle responses to the *Daphnia* delivery during silent control trials were rare (see Figure 4a).

Trials started with the delivery by hand of two live *Daphnia* to each side of the tank. Ten seconds after the first *Daphnia* introduction, noise playback began for the prolonged-noise and the brief-noise treatments. The control treatment had the same speaker switched on, but playing a silent track. In the brief-noise treatment, the noise stopped after 10 s and was followed by silent playback until the end of the trial. In the prolonged-noise treatment, noise continued until the end of the trial. Live *Daphnia* were delivered to alternate sides of the tank at 20 s intervals throughout the trial, which always lasted 300 s.

Non-food items (tank debris and reflections on the tanks walls) occurred naturally in the tank environment. They were not systematically introduced or controlled in this study, but there is no reason to suspect that the quantity of non-food items would be biased between treatments: repeat trials on the same fish were conducted in the same tank (any between-tank variation in debris will not bias debris levels between treatments within a single tank), and treatment order was counterbalanced so any within-tank variation from one trial to the next will be balanced across treatments.

### (e) Measures and analyses

During each trial, we recorded the rate and duration of any general allostatic (stress-related) responses, along the continuum of mild to severe (startles, freezing, hiding), and the consequences for the total number of *Daphnia* eaten. Startle responses were defined as sudden high speed movements (as per [40], [41]), periods of freezing as cessation of normal activity in the open tank, and periods of hiding as cessation of normal activity while located behind the cylinder housing the companion fish, where the focal fish was partly or fully hidden behind some opaque adhesive tape. We also recorded: the number of attacks towards food items (typical suction feeding mechanism with binocular fixation, movement towards prey and expansion of the buccal cavity); the number of attacks towards non-food items (attack movement typical of feeding, but directed towards non-food items); and the number of food-handling errors (occasions when a food item was attacked, but not successfully sucked into the mouth, or when food items were spat out and not recaptured). This allowed us to calculate more subtle measurements of foraging performance: food discrimination errors ( = non-food items attacked/(non-food items attacked + food items attacked)) and the resulting foraging efficiency, with respect to the foraging effort required to consume a given food intake ( = consumed items/(non-food items attacked + food items attacked)).

Analysis of variance of a repeated-measures linear model was used to examine the effect of playback treatment on each response (R version 2.8.1 [42]; core ‘stats’ package, ‘lm’ function with sequential sum of squares and normal/Gaussian error, model assumptions confirmed by visual inspection of residual plots, ‘anova’ function to assess significance of treatment factor). Linear models for three response variables were unbalanced due to missing data: one stickleback did not attack any food items during the brief-noise trial and thus had missing data for food-handling errors; a second stickleback did not attack any food items or non-food items when exposed to prolonged noise, and so had missing data for food-handling errors, food/non-food discrimination errors, and overall foraging. Substituting the average value per fish for the missing values gave the same results as the standard linear models (F ratios differing by <0.2), as did analysis using linear models robust to small numbers of missing values (‘lme4’ package [43], ‘lmer’ function with REML fit, significance of treatment factor examined by ‘anova’ comparing the standard model with a model where treatment effect is removed). The results from standard linear model analysis (with missing data for three variables) are therefore statistically robust, and are reported here.

## Results

Startle responses occurred significantly more frequently when either brief or prolonged noise was added to the environment compared to the silent control (F_2,46_ = 13.74, p<0.001; Figure 4a). However, noise had no detectable effect on the time spent frozen (F_2,46_ = 0.21, p = 0.811) or hidden (F_2,46_ = 0.71, p = 0.499). There was, therefore, no significant difference between trials in the total number of food items consumed (F_2,46_ = 0.67, p = 0.518; Figure 4b).

**Figure 5:**
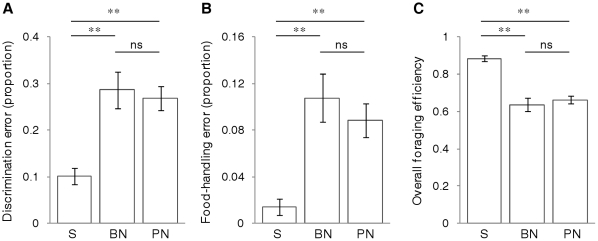
Acoustic noise increases foraging performance errors and reduces foraging efficiency. Response of foraging sticklebacks to playbacks of silence (S), brief (10 s) white noise (BN) and prolonged (300 s) white noise (PN). Bars show mean±1s.e.m. response for 24 fish during each playback of a repeated-measures experiment, with significant (**p≤0.01) and non-significant (ns p≥0.05) posthoc differences indicated (paired t-tests with Bonferroni correction). Brief noise and prolonged noise both significantly affected (A) the proportion of attacks towards non-food items, (B) the proportion of attacked food items that were not consumed, and (C) overall foraging efficiency (consumed items as proportion of all attacks on food and non-food items).

Foraging performance errors increased in response to both noise playbacks compared to the silent control. Food versus non-food discrimination was significantly poorer (F_2,45_ = 11.11, p<0.001; Figure 5a), driven by an increase in attacks on non-food items (F_2,46_ = 11.69, p<0.001; mean number of attacks ±1s.e.m.: brief noise  =  6.3±0.7, prolonged noise  =  7.1±1.1, silent control  =  2.3±0.4). Food-handling errors were also significantly more frequent (F_2,44_ = 11.90, p<0.001; Figure 5b), driven primarily by an increase in occasions that attacked food items were not successfully captured (F_2,46_ = 5.74, p = 0.006; mean number of missed attacks ±1s.e.m.: brief noise  = 1.2±0.4, prolonged noise  = 1.1±0.2, silent control  = 0.1±0.1). Foraging efficiency was consequently significantly lower in both noise treatments compared to the silent control (F_2,45_ = 26.57, p<0.001); that is, a greater foraging effort was required to consume the same number of prey items in noisy conditions compared to the silent control (Figure 5c).

## Discussion

Our results provide strong evidence that the addition of acoustic noise to an animal's environment can increase performance errors and therefore have a negative impact on foraging efficiency. Even 10 s exposure to white noise was sufficient to elicit this foraging disruption in sticklebacks, with no evidence that continuing the noise for 300 s led to any additional effects. Given that we found performance effects with even brief noise exposure, under rather benign testing conditions, and with a relatively simple task (animals were habituated to the environment, familiar with the prey, faced no competition, and were foraging in clear and well-illuminated waters where prey were easily visible and unable to escape the vicinity of the predator), we expect even greater potential impacts of noise under more realistic wild conditions.

Reduced food discrimination may have important indirect costs if non-food items are toxic or harmful in other ways, and reduced foraging efficiency may increase predation risk if individuals have to increase overall foraging activity to compensate [44]. If individuals persistently or frequently had to increase their effort to obtain the same food intake, net energetic gains may also decrease, with consequences for reproductive success and survival. Although animals might habituate to continuous exposure to the same noise [11], variable or unpredictable exposure or the occurrence of novel noise may prevent this (for example, three freshwater fish species show cortisol responses to variable boat noise but not to continuous Gaussian noise [12]) and could even sensitise them to such disturbances. The processes of habituation and sensitisation to noise exposure are only just beginning to be understood in any detail [45] and the implications are far from simple [46]. With rising concern over the potential impact of anthropogenic noise pollution on fish [47], examinations of the effects of more realistic temporal regimes of noise, including potential habituation or sensitisation to those that occur over a prolonged period, are a vital necessity.

A possible explanation for some of the foraging performance errors and the reduced foraging efficiency is that the addition of noise affects the behaviour of the *Daphnia* prey in some way - for example, increasing their alertness to the fish predator and thus affecting the fish foraging success - though this does not account for the noise-induced discrimination errors. More plausibly, the change in foraging behaviour may have been caused by a narrowing or shift of attention by the sticklebacks, because attention capacity is ultimately limited [27], [28]. The increase in food discrimination errors is similar to that seen in juvenile salmon (*Salmo salar*) when their need to attend to predator stimuli is increased [48], but the increase in attacks towards non-food items are incompatible with the narrowing of attention to specific stimuli that was evident in blue jays (*Cyanocitta cristata*) when attention demands were manipulated [49]. However, our documented reduction in foraging-task performance (reduced food/non-food discrimination and increased food-handling errors) and the increased number of attacks towards non-food items does support the hypothesis of a noise-induced attention shift; noise could potentially attract the attention of the fish, thus preventing them from focusing fully on the foraging task [25].

The possibility of noise-induced attention shifts is in line with Chan et al.'s [24] argument that noise can ‘distract’ animals from their primary task and detrimentally impact functionally important behaviour. The observed attention shift might also potentially be driven by a general allostatic response to a noise stressor, albeit a specific effect among a suite of more general changes in activity. Certainly, noise induced a greater frequency of startle responses among foraging fish: under control conditions, the appearance of the experimenter's hand when delivering *Daphnia* rarely induced a startle response, but during noise exposure and after the noise had ceased there was an increased sensitivity to such external stimuli. This may be simply the result of an increase in alertness under noisy conditions or evidence of a cognitive bias arising from noise-induced anxiety (whereby the fish treated the hand as a negative stimulus [50]). Although the increase in startle responses provides possible evidence for mild allostatic stress-related effects, the lack of any increase in freezing or hiding behaviour indicates that the impact of noise was much less than that generally seen in response to classic stressors such as predation risk (e.g. [20]). With no significant noise-induced cessation of foraging activity, it is perhaps not surprising that we also detected no reduction in absolute food intake when noise was added to the environment. Noise therefore had only a minor impact on the more obvious indicators of normal foraging activity.

There are two major implications arising from our results. First, the possibility that a wide range of cognitively influenced activities could be affected by noise, and thus that great care is needed when interpreting the behaviour of animals whenever they have been exposed to noise. Since these attention-mediated effects could occur in response to many different acoustic stimuli, and in the absence of stress-related effects, this implication will apply to a huge range of situations. As well as having welfare consequences, if normal performance or behaviour used to assess welfare needs (such as choice tests) are detrimentally affected [27], there will be potential impacts on scientific interpretation for the numerous behavioural studies conducted on captive animals in artificially loud enclosures. Second, with recent increases in scientific, public and political awareness of the potential problem of aquatic noise pollution, and a demand for more studies on behavioural impacts in fish in particular [2], [33], we provide timely evidence that noise pollution might have important effects on foraging behaviour even under the most benign of conditions.

Our study suggests that acoustic noise might influence a whole host of everyday activities through effects on attention. It also highlights the benefit of examining not only the more obvious immediate impacts of noise on behaviour, but also the more subtle effects that nonetheless have important implications and could occur in response to a broad range of acoustic stimuli. Future research is needed to elucidate the consequences of realistic noise regimes on wild animal behaviour, with consideration of habituation and sensitisation effects, but we demonstrate the potential for even very brief noise exposure to cause almost immediate effects that are functionally significant and that can persist beyond the cessation of the noise stimulus.
